# Detection of axillary lymph node metastasis in breast cancer using dual-layer spectral computed tomography

**DOI:** 10.3389/fonc.2022.967655

**Published:** 2022-10-10

**Authors:** Huijun Li, Huan Wang, Fangfang Chen, Lei Gao, Yurong Zhou, Zhou Zhou, Jinbai Huang, Liying Xu

**Affiliations:** ^1^ Department of Medical Imaging, School of Medicine, Yangtze University, Jingzhou, China; ^2^ Department of Radiology, Zhongnan Hospital of Wuhan University, Wuhan, China; ^3^ Department of Breast Surgery, Zhongnan Hospital of Wuhan University, Wuhan, China; ^4^ Department of Positron Emission Tomography/Computed Tomography (PET/CT) Center, The First Affiliated Hospital of Yangtze University, Jingzhou, China

**Keywords:** breast cancer, axillary lymph node, metastasis, dual-layer spectral detector computed tomography, DLCT

## Abstract

**Purpose:**

To investigate the value of contrast-enhanced dual-layer spectral computed tomography (DLCT) in the detection of axillary lymph node (ALN) metastasis in breast cancer.

**Materials and Methods:**

In this prospective study, 31 females with breast cancer underwent contrast-enhanced DLCT from August 2019 to June 2020. All ALNs were confirmed by postoperative histology. Spectral quantitative parameters, including *λ*_HU_ (in Hounsfield units per kiloelectron-volt), nIC (normalized iodine concentration), and Z_eff_ (Z-effective value) in both arterial and delay phases, were calculated and contrasted between metastatic and nonmetastatic ALNs using the McNemar test. Discriminating performance from metastatic and nonmetastatic ALNs was analyzed using receiver operating characteristic curves.

**Results:**

In total, 132 ALNs (52 metastatic and 80 nonmetastatic) were successfully matched between surgical labels and preoperative labels on DLCT images. All spectral quantitative parameters (*λ_Hu_*, nIC, and Z_eff_) derived from both arterial and delayed phases were greater in metastatic ALNs than in nonmetastatic SLNs (all *p* < 0.001). Logistic regression analyses showed that *λ_Hu_* in the delayed phase was the best single parameter for the detection of metastatic ALNs on a per-lymph node basis, with an area under the curve of 0.93, accuracy of 86.4% (114/132), sensitivity of 92.3% (48/52), and specificity of 87.5% (70/80).

**Conclusion:**

The spectral quantitative parameters derived from contrast-enhanced DLCT, such as *λ_Hu_*, can be applied for the preoperative detection of ALN metastasis in breast cancer.

## Introduction

Breast cancer is one of the most pervasive cancer types and the second deadliest malignancy in women (1, 2). A key prognostic factor in early breast cancer is axillary lymph node (ALN) status (3–7). Accurate detection of ALN status is thus critically important for the management of patients with breast cancer. Sentinel lymph node (SLN) biopsy has emerged as a substitute for ALN dissection and a probe for ALN staging (8–10). SLN biopsy, however, is an invasive procedure associated with a risk of nerve damage and hematoma formation, and the accurate localization of target nodes can be challenging (6, 11). Imaging evaluations may serve as a noninvasive alternative approach to lymph node staging.

Ultrasonography has been shown to be effective in predicting ALN metastasis with the morphological and spatial features of target lymph nodes, although the reliability of this approach is limited by the sternum and costal cartilage (12, 13). Structural and functional magnetic resonance imaging (MRI) facilitates more accurate detection of metastatic lymph nodes, yielding greater specificity than ultrasonography, with better consistency across pathological findings (14). Relative to SLN biopsy, however, MRI is significantly less sensitive and can yield false-negative results (15, 16). Positron emission tomography/computed tomography (PET/CT) imaging incorporates both anatomical and molecular functional imaging modalities to predominate the qualitative restrictions of CT scanning and the potential for false-positive results in PET scans due to physiological uptake. Notably, PET/CT scanning is not impacted by dense breast tissue, scarring, or prostheses, and it is better suited to ALN metastasis detection in breast cancer than more conventional imaging approaches (17). Owing to its high cost, however, PET/CT is not well suited for routine preoperative use.

Although computed tomography (CT) is not a primary modality for the evaluation of breast masses and ALN, it has been used widely in preoperative staging to assess whether the patient has lung metastasis. Contrast-enhanced conventional CT has difficulty identifying some metastatic lymph nodes without morphological abnormalities. Spectral CT is being increasingly used and has been shown to add value in oncological imaging (18–20). The high- and low-energy data collected by the DLCT are analyzed on the premise of complete matching in time and space in the projection data domain and then stored in the host, workstation or image storage and transmission system in the form of holographic spectral image (SBI) base data packets. DLCT imaging performed using a two-layer detector can offer multiparameter insights into tissue characteristics that cannot be obtained through conventional CT imaging. This approach offers several advantages, including improved sensitivity, the ability to detect lesions with qualitative accuracy, the ability to assess the composition of specific substances, and a reduction in metal-induced imaging artifacts (21). DLCT energy imaging has been reportedly used to evaluate patients with cardiovascular diseases, abdominal parenchymal organ tumors, biliary tract lesions, and gastrointestinal diseases (22–25). The value of preoperatively diagnosing ALN metastases in patients suffering from breast cancer, however, remains to be established.

Therefore, we considered whether contrast-enhanced DLCT could detect ALN metastases more accurately. To our knowledge, no previous study has used contrast-enhanced DLCT in detecting ALN metastases in breast cancer patients. The objective of this study was to assess the diagnostic performance of spectral quantitative parameters of DLCT in the preoperative detection of ALN metastasis in patients with breast cancer.

## Materials and methods

### Participants

This was a prospective, single-center study that received approval from the ethics committee of Zhongnan Hospital, Wuhan University. All participants provided written informed consent before enrollment in this study.

**Figure 1:**
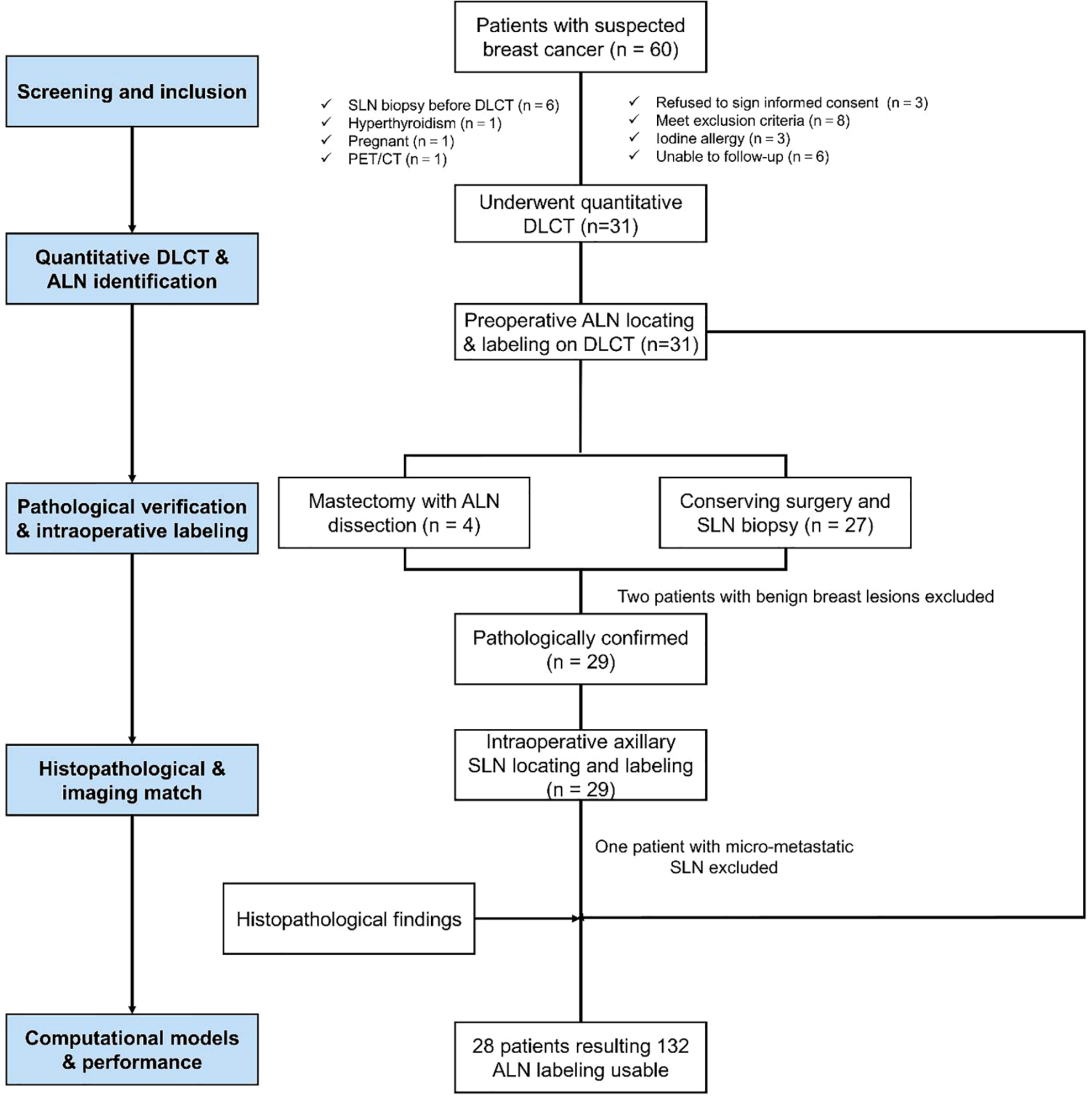
The flow chart shows the enrollment of participants with breast cancer in this study. SLN, sentinel lymph node.

In total, 60 patients with suspected breast cancer from August 2019 to June 2020 were recruited from Zhongnan Hospital, and 31 patients underwent conventional and contrast-enhanced chest DLCT examinations (conventional, arterial, and venous phases) before any treatment. The inclusion criteria were participants who: (1) were confirmed to have breast cancer by postoperative pathology; (2) had not undergone radiotherapeutic, chemotherapeutic, endocrine, targeted, or other treatments; (3) had no history of other neck or chest tumors; and (4) were free of hyperthyroidism, renal failure, or serious cardiopulmonary disease. Participants were excluded if they did not meet the inclusion criteria and were lost to follow-up. Overall, 28 patients diagnosed with breast cancer were included in the final analyses. The flow chart shows the enrollment of participants (**Figure 1**).

### DLCT scanning

All participants underwent chest contrast-enhanced CT with DLCT (IQon SpectralCT, Philips Healthcare, Best, the Netherlands) in the prone position for the duration of scanning, with the chest secured with matching foam pads, their hands placed on their sides and their breasts hung naturally. The prone position (26) was used as it is better suited to detecting breast lesions without interfering with the evaluation of the lymph nodes of the axillary and supraclavicular regions. After conventional DLCT scanning, arterial phase and delayed phase scanning were performed. The scanning parameters of conventional DLCT were slice thickness = 2 mm, collimation = 64*0.625 mm, slice increment = 2 mm, pitch = 0.609, 120 kVp, and automated tube current modulation. For contrast-enhanced DLCT, a monomeric nonionic contrast agent (Iopamiro; 370 mg/dL iodine, Bracco) was injected into the vein of the participants’ contralateral arm (CT Contrast Injector, CT motion, XD8000). The total contrast dose was 1.5 times per kilogram of each participant’s weight, adding 25 ml of normal saline, and the flow rate was 2-2.5 mL/s. Data acquisition time: arterial phase delay 10 s after trigger, with a threshold value of 150 HU, trigger at aortic arch. Delayed phase: delay 90 s after contrast agent injection. These scanning parameters were selected based upon a previous article on the differentiation between benign and malignant breast cancers (27).

### Preoperative localization and intraoperative node identification

**Figure 2:**
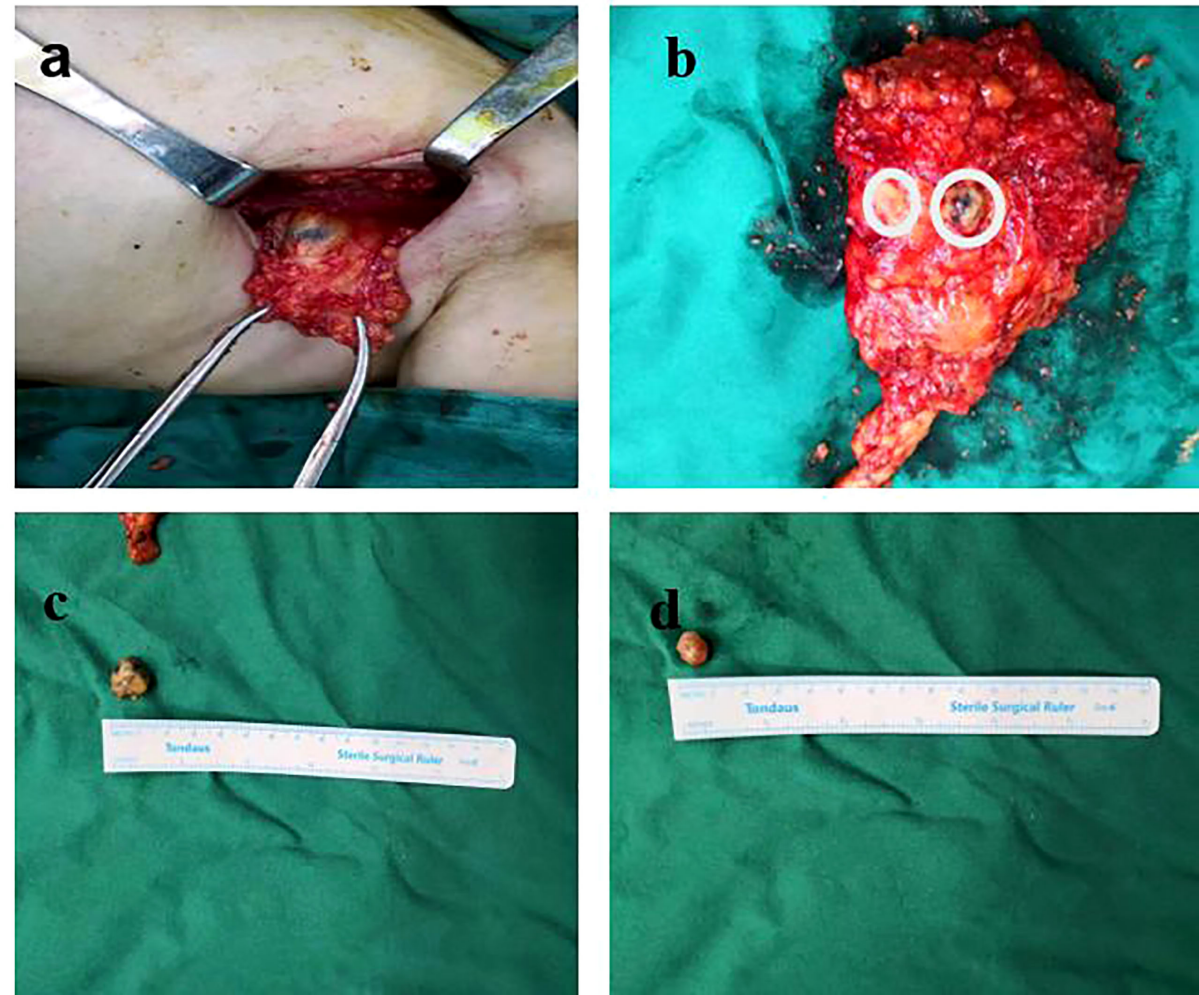
Lymph nodes were resected according to the order of the preoperative labeling on DLCT images. **(A)** Labeled lymph nodes of each participant were evaluated by the same breast surgeon who had conducted the surgical resection of those labeled lymph nodes. **(B)** The intact axillary tissue and related lymph nodes were carefully retained. **(C, D)** The removed lymph nodes were measured and numbered, and communication was maintained with the pathologist.

Lymph nodes were identified based on the preoperative labeled DLCT images and were matched based on their shape, size, and adjacency to other nodes. Preoperatively, radiologists sequentially labeled all ALNs from central and lateral, anterior and posterior, and apical lymph nodes on enhanced DLCT images. Labeled lymph nodes of each participant were evaluated by the same breast surgeon who had conducted the surgical resection of those labeled lymph nodes. The intact axillary tissue and related lymph nodes were carefully retained. Communication was also maintained with the doctor who sent the final pathological results, with the relevant lymph nodes being marked appropriately to facilitate comparisons with these pathological findings (**Figure 2**).

### Quantitative parameters of ALNs

**Figure 3:**
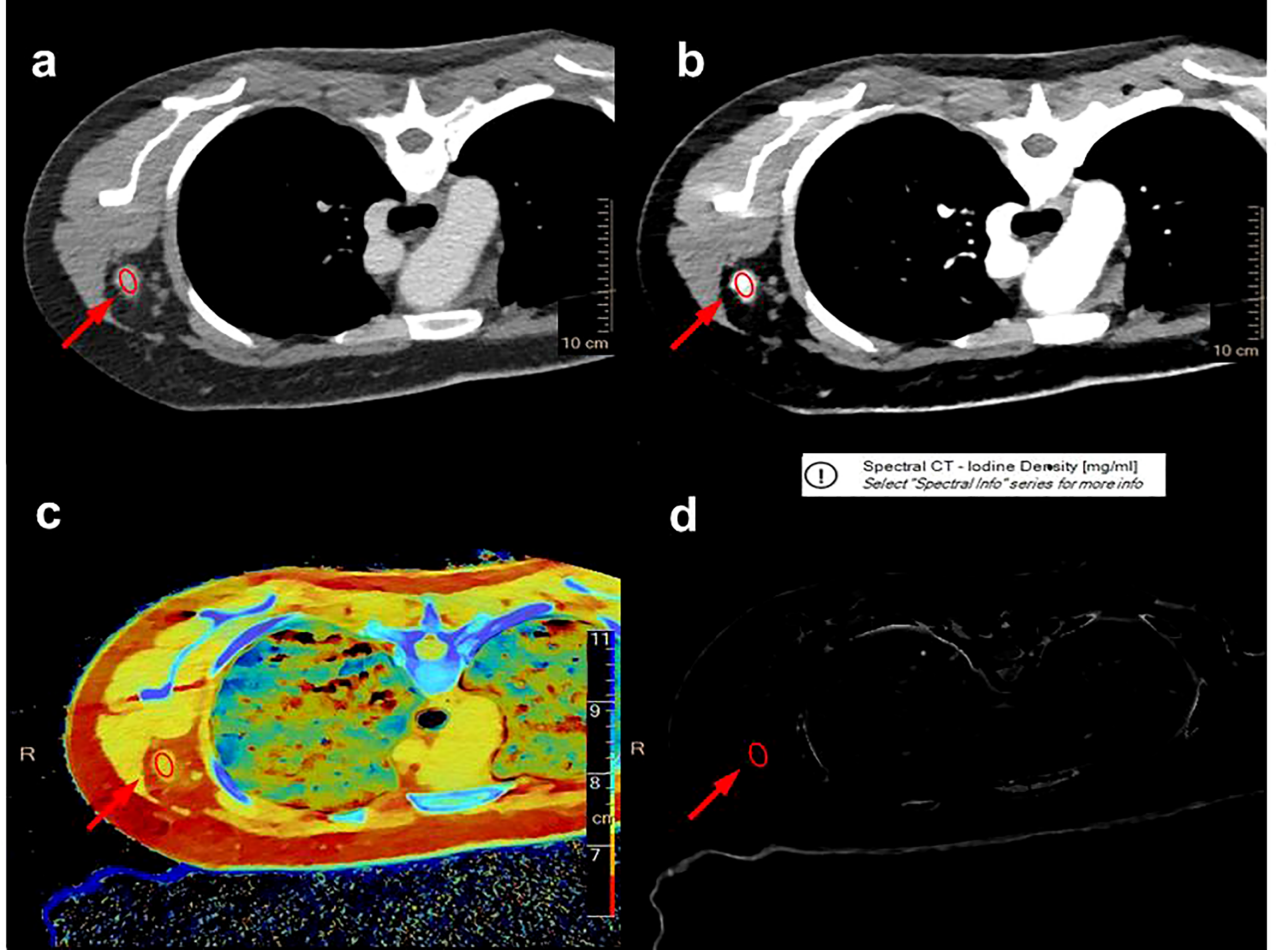
A multiplane reconstruction technique was used to place the ROI to cover the entire axillary lymph node (red ellipse) on the IQon 40 keV **(A)** and 70 keV **(B)** DLCT images, and quantitative parameters, such as effective atomic number **(C)** and iodine concentration **(D)**, were recorded in the arterial and venous phases, respectively.

**Figure 4:**
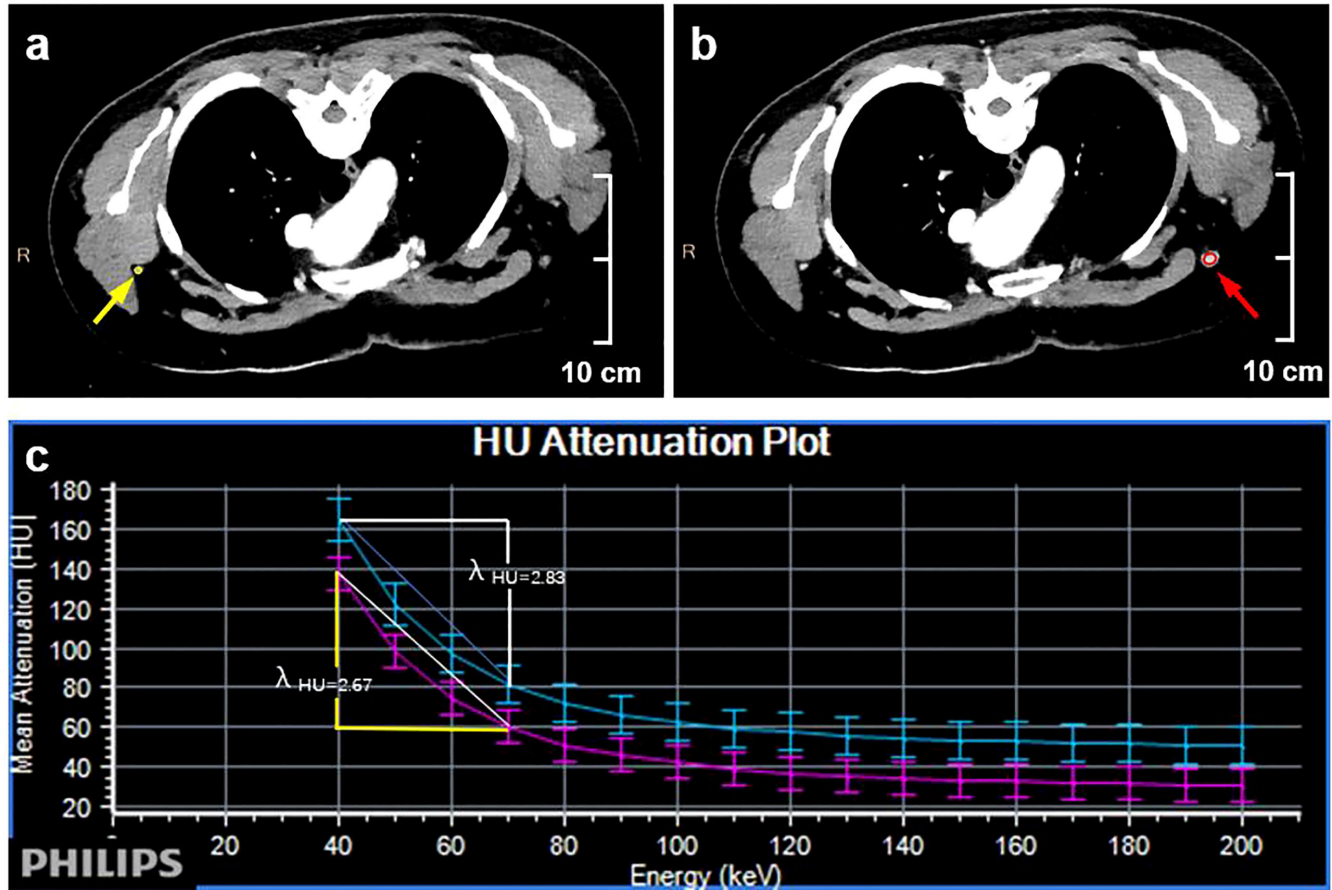
Quantitative dual-layer spectral CT parameter measurement of the same patient as in **Figure 3**. **(A)** The ovoid area of interest (yellow) is shown on a 70 keV enhanced image of a nonmetastatic lymph node. **(B)** The oval-shaped region of interest (red) is shown on the 70 keV enhanced image of the metastatic lymph node. **(C)** The graph shows that the value of *λ_Hu_* is higher in the metastatic lymph node than in the nonmetastatic lymph node (2.83 >2.67).

Two radiologists with a decade of experience in breast imaging diagnosis independently evaluated all imaging results in this study, both of whom were blinded to the pathological results. A multiplane reconstruction technique in the Philips workstation was used to place a region of interest (ROI) that covered the entirety of a given ALN (**Figure 3**) on IQ on 40 keV and 70 keV images, and quantitative parameters, including CT value, iodine concentration, and effective atomic number, were measured in the arterial and venous phases. A 20-mm ROI was placed on the aorta to measure the iodine concentration and effective atomic number, and the normalized iodine concentration (nIC) was then calculated by dividing the iodine concentration in the ALN by the iodine concentration in the aorta. Similarly, the normalized effective atomic number (nZ_eff_) was measured by dividing the effective atomic number of the SLN through that of the aorta. The slope of the spectral Hounsfield unit curve (*λ_Hu_*) was assessed by subtracting the value of CT derived from a given 40 keV image from the CT value derived from a given 70 keV image divided by 30(**Figure 4**). The 40 keV and 70 keV arterial and venous CT values, iodine concentration, effective atomic number, *λ_Hu_*, nIC, nZ_eff_, and other quantitative parameters were established for each lymph node. Scanning data (*λ_Hu_*, nIC, nZ_eff_) were stored for processing and analysis.

### Statistical analysis

The Shapiro‒Wilk test was used to assess the normality of the quantitative DLCT parameters. Intraclass correlation coefficients were used to ensure test-retest reliability when evaluating the quantitative evaluation results from two different radiologists. Continuous variables were meticulously studied for either metastatic or nonmetastatic ALNs using Student’s t tests. The ability of individual CT parameters to differentiate between metastatic and nonmetastatic ALNs was assessed through univariable binary logistic regression analyses, with those parameters achieving a *P* < 0.05 being incorporated into a subsequent multivariable binary logistic regression analysis. For each lymph node, a 95% confidence interval (CI) was calculated. The curves of the received operatic characteristic (ROC) were implemented to predict ALN metastases, with sensitivity, accuracy, and specificity calculated. Optimal threshold values were established based upon Youden’s index, and SPSS was used for all statistical testing.

## Results

Thirty-one patients underwent DLCT with available histopathological reports, and 28 had confirmed breast cancer. Of the 28 patients, including 2 patients with bilateral breast cancer, 132 ALNs were matched successfully between surgical labels and preoperative labels on DLCT images. According to the pathological results, 52 metastatic and 80 nonmetastatic ALNs were confirmed. The diagnostic accuracy of axillae and patients was 86.7% (26/30) and 89.3% (25/28), respectively. The pathological statistics are shown in **Table 1**.

**Table 1:** Patient demographics and clinical data.

		Number	Percentage (%)
**Demographics**			
Patients included		28	–
Axillae		30	100%
Age (years)		53.3 ± 10.7	–
Childbearing history			
	Yes	27	96.4%
	No	1	3.6%
Menstruation		25	89.29%
	Premenopause	11	39.3%
	Menopause	17	60.7%
**Clinical data**			
Family history		11	
Side (R/L/bilateral)		11/15/2	
Patient history			
	Primary breast cancer	26	92.86%
	Recurrent ipsilateral breast cancer	2	7.14%
Type of breast cancer			
	Invasive ductal carcinoma	25	89.29%
	Invasive lobular carcinoma	2	7.14%
	Mixed	1	3.57%
Diagnostic accuracy of axillae		26	86.7%
Diagnostic accuracy of patients		25	89.3%
Estrogen receptor-positive tumor		16	57.14%
HER2-positive tumor		7	25.0%
Previous ipsilateral radiotherapy		6	21.43%
Previous adjuvant chemotherapy		6	21.43%
SLN biopsy			
	Average number of SLN	3.8	
	Metastatic	15	57.7%
	Nonmetastatic	11	42.3%
ALN dissection (Metastatic/Nonmetastatic)		4/0	
pT1N0		7	23.3%
pT1N1-3		10	33.3%
pT2N0		3	10.0%
pT2N1-3		4	13.3%
pT3N0		1	3.3%
pT3N1-3		3	10.0%
pT4N0		0	0.0%
pT4N1-3		2	6.67%

We found that DLCT parameters, including *λ_Hu_*, nIC, and nZ_eff_, in the arterial phase and the venous phase were all considerably greater in metastatic axillary lymph nodes than in nonmetastatic nodes (*p* ≤ 0.001).

**Figure 5:**
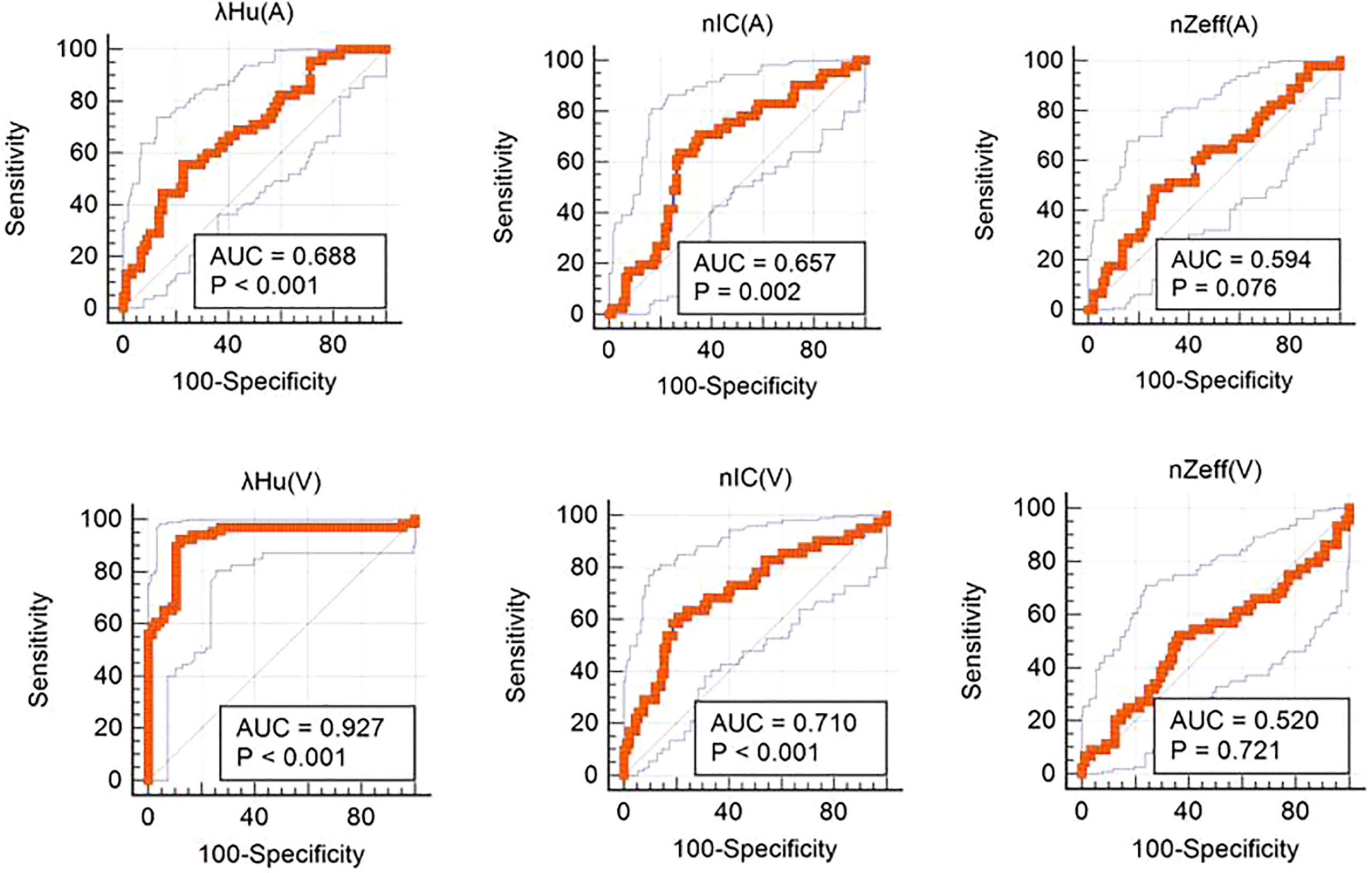
Receiver operating characteristic curves of quantitative dual-layer spectral computed tomography parameters for differentiating metastatic and nonmetastatic axillary lymph nodes. AUC, area under the curve.

The analyses of multivariate logistic regression illustrated that venous phase *λ_Hu_* was the most reliable predictor of axillary lymph node metastasis among these tested parameters. ROC curve analyses indicated that *λ_Hu_* exhibited the highest differential diagnostic utility in the venous phase, with an accuracy rate of 86.4% (114/132) [95% CI: 80.5%-92.2%], a sensitivity of 92.3% (48/52) [95% CI: 83.2%- 97.5%], and a specificity of 87.5% (70/80) [95% CI: 77.5%- 94.6%]. The accuracy, sensitivity, and specificity of *λ_Hu_* in the venous phase were higher than those for other parameters (*p* < 0.001) (**Table 2**; **Figure 5**).

**Table 2:** The receiver operating characteristic curves of the metastatic and non-metastatic lymph nodes were analyzed using the quantitative parameters of quantitative dual-layer spectral CT.

	AUC	Sensitivity (%)	Specificity (%)	Accuracy (%)
λ_Hu_ (A)	0.68	55.56(29/52) [40.0-70.4]	77.01(62/80) [66.8-85.4]	69.7(92/132) [61.9-77.5]
λ_Hu_ (V)	0.93	92.42(48/52) [83.2-97.5]	87.88(70/80) [77.5-94.6]	86.4(114/132) [80.5-92.2]
nIC (A)	0.66	63.41(33/52) [46.9-77.9]	72.53(58/80) [62.2-81.4]	62.9(83/132) [54.6-71.1]
nIC (V)	0.71	60.98(30/52) [44.5-75.8]	79.12(64/80) [69.3-86.9]	68.2(90/132) [60.2-76.1]
nZ_eff_ (A)	0.59	48.89(25/52) [33.7-64.2]	73.56(59/80) [63.0-82.4]	54.5(72/132) [46.1-63.0]
nZ_eff_ (V)	0.52	52.27(27/52) [36.7-67.5]	63.64(51/80) [52.7-73.6]	62.9(83/132) [54.6-71.1]

Note. —Data in parentheses are the numerators/denominators, and data in brackets are 95% confidence intervals. The unit for the threshold of parameter for arterial phase λ_Hu_ and venous phase λ_Hu_ is Hounsfield unit per kiloelectron-volt. AUC = area under the curve, λ_Hu_ = slope of the spectral Hounsfield unit curve, nIC = normalized iodine concentration, nZ_eff_ = normalized effective atomic number.

## Discussion

Imaging approaches to preoperatively evaluate breast cancer patients for axillary lymph node metastasis include ultrasound, MRI, CT, and PET/CT approaches. Ultrasonography offers the advantages of avoiding radiation and enabling the real-time visualization of lesion features, which may aid in the detection of metastatic nodes. However, the sensitivity, specificity, positive predictive, and negative predictive values of ultrasound-based approaches are only 35.8%, 92.8%, 68.7%, and 76.6%, respectively. An et al. (28) compared the relative diagnostic utility of PET/CT, ultrasonography, and MRI for axillary lymph node staging in breast cancer patients and found sensitivity and specificity values of 79.2% and 74%, respectively, for lymph node metastasis, with these values being lower than those associated with the best IQ on CT parameters. In this case, the evaluation of the multiparameter information contained in the spectral CT dataset can further clearly distinguish axillary and extraxillary lymph node metastasis, thus making it possible to perform more accurate N staging for breast cancer patients.

To our knowledge, our study group is the first to compare the spectral CT data of benign and malignant lesions in axillary lymph nodes of breast cancer and the only one to use a multiparameter quantitative method (including *λ_Hu_*, nIC, and Z_eff_). As the main result, we demonstrate the derivation of quantitative data. The results show that contrast-enhanced multiparameter CT can predict axillary lymph node metastasis.

In our study, among IQon CT quantitative parameters, the slope of energy spectrum curves (λ_HU_) in arterial and venous phases, the normalized iodine concentration (nIC) in arterial and venous phases, and the effective atomic number of axillary lymph nodes in arterial and venous phases were significantly higher than those in nonmetastatic axillary lymph nodes (*P ≤* 0.001). The diagnostic accuracy of axillae and patients was 86.7% (26/30) and 89.3% (25/28), respectively. The accuracy of λ_HU_ in detecting axillary lymph node metastasis at the venous stage was 86.4% (114/132), and the sensitivity was 92.3% (48/52). The specificity was 87.5% (70/80), and the AUC value was 0.93. Multivariate logistic regression analysis showed that λ_HU_ was the best single parameter for the detection of axillary lymph node metastasis at the venous stage, with high accuracy, and had high reference value for the preoperative prediction of axillary lymph node metastasis. The conclusion of our study is consistent with the results of Zhang et al. (29). The spectral detector CT parameter λ_HU_ at the venous stage has a strong ability to preoperatively diagnose axillary lymph node metastasis in breast cancer patients, and it is higher than conventional morphological parameters. λ_HU_ can provide quantitative information on tissue components. The components in metastatic lymph nodes with tumor cell infiltration are different from those in normal lymph nodes, resulting in different spectral curves, which overcomes the limitation of single-energy CT imaging based on energy attenuation (30–32). However, some studies (20) showed that the spectral parameter values of metastatic axillary lymph nodes were all lower than those of nonmetastatic axillary lymph nodes, which was inconsistent with our study. The reason may be related to the selected cases. We enrolled breast cancer patients with axillary lymph node metastasis for the study, while the above study analyzed the spectral parameters with mediastinal lymph node metastasis of lung cancer. The low measured value of inflammatory nodules in lung cancer can be explained by factors such as low vascular permeability and nonobvious vascular dilation. λ_HU_ has high accuracy and can be used as an auxiliary method for the preoperative identification of axillary lymph node metastasis in breast cancer patients, helping surgeons avoid subjective bias due to lack of experience and improving the reliability of diagnosis.

In our study, the spectral parameter nIC was used as one of the parameters for the quantitative evaluation of axillary lymph node metastasis of breast cancer; the AUC value of the venous phase was 0.71, the sensitivity was 60.9%, the specificity was 79.1%, and the accuracy was 68.2%. The AUC value of nIC in the arterial phase was 0.66, with a sensitivity of 63.4%, specificity of 72.5% and accuracy of 62.9%, which can be used as a quantitative parameter to identify axillary lymph node metastasis of breast cancer. In this study, the nIC value in the metastatic axillary lymph node group was higher than that in the nonmetastatic axillary lymph node group. Simon et al. (20) evaluated lymph node metastasis in prostate cancer patients with dual-layer spectral detector CT and found that the spectral parameter nIC value of metastatic lymph nodes was higher than that of nonmetastatic lymph nodes (1.9 ± 0.6 mg/mL vs. 1.5 ± 0.5 mg/mL, *P*< 0.05); the AUC value was 0.72, the sensitivity was 81.3%, and the specificity was 58.5%. The sensitivity and specificity were 12.8% and 99.0%, respectively, when the diameter of the short axis was 1 cm. The sensitivity of this study was low, and the reason for the slight difference is mainly due to the different research objects. Our study is mainly about breast cancer, while Simon’s study is about prostate cancer patients. The composition of lesions is different; thus, inevitable data differences may explain the conclusions. It has been proven that (33, 34) nIC can distinguish soft tissue according to the difference in iodine and soft tissue attenuation at different energy levels. Iodine density mapping combined with CT signs performed well in the diagnosis of cervical lymph node metastasis of thyroid cancer, and the AUC value in the validation dataset was 0.895 (35). Volterrani et al. (36) found that the concentration of iodine in axillary lymph node metastases was higher than that in nonmetastatic lymph nodes. Taking a 0.5 mg/mL iodine concentration as the dividing point between lymph node metastasis with suspicious morphology and nonmetastatic lymph nodes, the sensitivity of dual-layer spectral detector CT could be improved. The calculation of standardized iodine concentration can exclude the influence of systemic circulation, blood vessels and other factors between different individuals and reflect local blood supply more accurately and objectively. NIC has potential clinical value in the field of breast cancer axillary lymph nodes.

There are several limitations in this study. First, there might have been a sample bias because the positive rate of SLNB in this study was approximately 57.7% (15/26), slightly higher than that in other studies, which may have led to the higher accuracy of preoperative DLCT multiple parameter analysis in assessing axillary lymph node metastasis. In fact, the main purpose of this study was to verify the feasibility and applicability of the DLCT multiparameter evaluation method; thus, it does not affect the conclusion of this study. The second limitation might be that we performed DLCT scans, including the arterial phase and venous phase, which may have increased the radiation risk of patients. We introduced this situation in detail in the informed consent form and obtained the consent of patients. Moreover, the average age of patients in this group was more than 50 years old, and they can theoretically tolerate the increased radiation dose. Third, we only assessed axillary lymph nodes that were preoperatively labeled on DLCT images, whereas unlabeled nodes were not included in this study.

## Conclusion

In summary, our study determined that *λ*_HU_ derived from DLCT in the venous phase was the optimal quantitative spectral parameter for detecting ALN metastasis in patients with breast cancer, highlighting a noninvasive approach for the preoperative prediction of ALN metastasis and opening new avenues for the management and treatment of patients with breast cancer.

## Data availability statement

The original contributions presented in the study are included in the article/Supplementary Material. Further inquiries can be directed to the corresponding authors.

## Ethics statement

Written informed consent was obtained from the individual(s) for the publication of any potentially identifiable images or data included in this article.

## Author contributions

HL: Investigation, Methodology, Formal Analysis, Software, Writing-Original Draft; HW: Data Collection, Methodology, Writing-Original Draft;FC: Investigation, Data Collection, Methodology; LG: Supervision, Writing-Review and Editing; YZ and ZZ: Data Collection, Software; JH: Conceptualization, Investigation, Methodology, Supervision; LX: Conceptualization, Funding Acquisition, Resources, Supervision, Writing-Review and Editing. All authors contributed to the article and approved the submitted version.

## Funding

This study was supported by the National Natural Science Foundation of China (82172055) and Clinical research and development project of Science and Technology Innovation Cultivation Fund of Zhongnan Hospital of Wuhan University (Grant No. lcyf202109).

## Acknowledgments

We thank the study participants and referring technicians for their participation in this study.

## Conflict of interest

The authors declare that the research was conducted in the absence of any commercial or financial relationships that could be construed as a potential conflict of interest.

## Publisher’s note

All claims expressed in this article are solely those of the authors and do not necessarily represent those of their affiliated organizations, or those of the publisher, the editors and the reviewers. Any product that may be evaluated in this article, or claim that may be made by its manufacturer, is not guaranteed or endorsed by the publisher.
